# Relevance of small areas of myocardial ischemia in adenosine stress MR

**DOI:** 10.1186/1532-429X-16-S1-P197

**Published:** 2014-01-16

**Authors:** Lorenzo Monti, Fabio Longaretti, Maria Pia Del corral, Veronica Lisignoli, Barbara Nardi, Luca Balzarini

**Affiliations:** 1 Radiology, Humanitas Research Hospital, Rozzano(MI), Italy; 2 Cardiology, Humanitas Research Hospital, Rozzano, Italy

## Background

We sought to compare the clinical relevance of reporting all the areas of ischemia observed at visual interpretation of stress perfusion ("radiologic" approach: method 1), versus the hypothesis of reporting only mild-to-moderate areas of ischemia, about 8 to 10% or more of LV mass ("cardiologic" approach: method 2).

## Methods

Retrospective re-analysis of a series of 283 stress MR, with perfusion series acquired at 4' of adenosine infusion at 140 mcg/Kg/min, 3 slices every heart beat, allowing a 16-segments myocardial segmentation. MR adenosine stress was reported as positive: in method 1 in presence of any area of subendocardial perfusion defect, and in method 2 only if at least 3 subendocardial regions (3 half segments in the 16 segments model).

## Results

We found 75 patients with a coronary angiography < 2 months after the diagnostic test. Patients with a negative stressMR were excluded in the absence of a coronary angiography. Prevalence of disease among the studied population was 73,3%. Method 1: sensitivity 89,1%, specificity 45%, PPV 81,6% (95%CI:70 to 89); NPV 60% (95% CI 35.7 to 80,2), global accuracy of 77,33%. Method 2: sensitivity78,2%, specificity 75%, PPV 89,6% (95% CI 77,8 to 95,5), NPV (95% CI 37,3 to 72,4), global accuracy of 73.3%.

## Conclusions

Exclusion of small areas of ischemia doubled the number of false negative results from 6 to 12. The global accuracy of the exam did not change between the 2 reporting methods. Therefore, also small areas of myocardial ischemia should be reported in a stress MR report.

## Funding

None.

